# Sources of Conflict and Prevention Proposals in User Violence Toward Primary Care Staff: A Qualitative Study of the Perception of Professionals

**DOI:** 10.3389/fpubh.2022.862896

**Published:** 2022-06-15

**Authors:** David Pina, Carmen María Peñalver-Monteagudo, José Antonio Ruiz-Hernández, José Antonio Rabadán-García, Paloma López-Ros, Begoña Martínez-Jarreta

**Affiliations:** ^1^Department of Socio-Health Sciences, Faculty of Medicine, University of Murcia, Murcia, Spain; ^2^Applied Psychology Service, University of Murcia, Murcia, Spain; ^3^Department of Psychiatry and Social Psychology, Faculty of Psychology, University of Murcia, Murcia, Spain; ^4^Department of Behavioral Sciences and Health, University Miguel Hernández, Elche, Spain; ^5^Department of Pathological Anatomy, Forensic and Legal Medicine and Toxicology, University of Zaragoza, Zaragoza, Spain

**Keywords:** health care violence, user violence, sources of conflict, prevention proposal, qualitative study

## Abstract

**Background:**

Some studies indicate that at least one in four cases of workplace violence occurs in the health sector, with a higher incidence in Emergency Departments, Mental Health Services or Primary Care. Unlike other professional groups, healthcare workers perceive this type of behavior mainly from users or patients. This is the reason why both the detection of conflict between users and professionals and the ways to face and reduce these conflicts has been and is one of the main fields of study in this population. The aim of this study was to delve into the sources of conflict between users and professionals in Primary Care from the perspective of the professionals themselves. In addition, the aim was to explore the proposals for intervention/prevention of this conflict that the professionals perceived as necessary to improve the work environment.

**Methods:**

This study uses qualitative methodology conducting 8 focus groups with professionals related to Primary Health Care. The final sample was composed of 44 workers who were part of the regional management, labor unions, area coordinators, center coordinators and representatives of the professional groups of these centers (medicine, nursing and administration). Thematic analysis was used to extract topics and subtopics.

**Results:**

The results are divided into areas of conflict and intervention proposals. The professionals detect a lack of training or education in themselves, absence of functional multidisciplinary teams or competencies to improve the patient-professional relationship, among others. To address these shortcomings, they propose the creation of protocols for action in the face of aggression, the formation of spaces and channels of communication both among the center's own workers and between them and other organizations (e.g., hospitals), fostering a positive relationship with the user community and ongoing training in various topics such as self-safety, management of emotions, empathy or interpersonal communication.

**Conclusions:**

This study allows to highlight specific areas of user-professional conflict in Primary Care. Furthermore, the inclusion of intervention proposals by the professionals allows to propose starting points for the development of complete plans.

## Introduction

Work, besides being a source of livelihood, is an important factor for our self-esteem, intellectual development, well-being and/or social relations (1). This is why workplace violence is a worldwide social problem of great interest both in terms of practical care and research (2). The International Labor Organization has defined this phenomenon as “any action, incident or behavior that departs from reasonable conduct in which a person is assaulted, threatened, harmed, injured in the course of, or as a direct result of, his or her work”. Thus, this definition includes different behaviors such as physical aggression, verbal abuse, intimidation, harassment, and sexual, racial or psychological harassment (3). The perpetrator of workplace violence can be either from outside the workplace (clients/patients and companions) or inside the workplace (supervisors and other co-workers) (4, 5). In general terms, there has been an exponential increase in workplace violence, with around 25% of professionals being exposed, and considering health service personnel, among all professionals, as most at risk of suffering violence (6–8). This situation has not improved since the emergence of COVID-19. Health centers have been subjected to a high demand and pressure for care, which can lead to distancing and the deterioration of the user-professional relationship. This new healthcare paradigm could be related to the increase in violence recently registered, predominantly non-physical violence and especially directed at medical and/or nursing staff (9–13).

In the health sector, violence from external sources comes mainly from users and their companions, with multiple consequences associated with the exposure to this violence (14, 15). This type of violence is bidirectional, meaning that user violence damages the professional-user relationship, which in turn can lead to greater conflict. Generally, exposure to workplace violence in this sector has been associated with a poorer quality of care, lower job performance, less commitment, greater negligence and greater deterioration in the cognitive functioning of workers, among others (16, 17). The factors that contribute to violence in the health care setting are varied and complex. Gender, diagnosis, symptomatology, environmental conditions, perception of poor communication, substance abuse, feelings of frustration, denial of services, overcrowded wards or staff training are some of the variables that the literature points out as relevant (18).

Recent studies show that workplace violence is widespread in all continents, ranging from 48.1% in Europe and 70.9% in Oceania (2, 8, 19). In the context of this study, the data on workplace violence are equally worrisome (20). A systematic review of workplace aggression has estimated that up to 83% of healthcare workers may have been the target of violent acts (21). Specifically, a higher prevalence of non-physical violence (42.5%) compared to physical violence (24.4%) has been observed, although physical violence in the Emergency department and Psychiatry is differentially higher than in other services (22). In general terms, the units at highest risk seem to be pre-hospital services (83.9%), emergencies (79.4%), Mental Health (68.1%) and Primary Care (PC) (50.7%). Regarding the professional category, there seems to be a similar risk between healthcare and non-healthcare personnel in these services, although there does not seem to be a consensus on this in the literature, with a greater risk sometimes being observed in medical or nursing personnel (2, 8, 19, 23–25).

As mentioned above, PC services have a high risk of user violence toward healthcare personnel. Most of the studies address this phenomenon from a quantitative perspective, concluding that, in general terms, all professionals are exposed to user violence, having consequences on job satisfaction, empathy, suffering from burnout or the reduction of the workers' psychological well-being (25–27). Especially, since the emergence of COVID-19, healthcare PC professionals have been working in environments that are overburdened with stress and, at times, understaffed and undersupplied. This could imply an increased risk of workplace conflict and, therefore, an increased risk to the psychological health of these workers (28).

From a qualitative perspective, there are hardly any studies evaluating user violence. A recent study with PC users concluded that they positively value the changes in health care aimed at improving waiting times, comfort and the agility of procedures and bureaucracy. At the same time, they consider a source of conflict the lack of information on waiting times, the therapeutic process or the impersonal treatment by healthcare personnel (29). In short, the bibliography concludes that both education and training in the prevention and reduction of aggression are key aspects to address this problem. In general terms, these studies agree on the need to develop competencies in the prevention and management of violence, being considered a requirement for the creation of services that can address the challenges related to aggression (30).

The described reality of violence in health care centers has motivated international organizations such as WHO, ILO, ICD or the OISP to create guidelines to promote violence-free institutions (13). In the context where this study takes place, there are similar initiatives such as the National Observatory on Violence against Doctors in 2010, the Working Group against Violence against Professionals in the National Health System in 2013 and, in 2018, the creation of the National Observatory on Violence against Nurses in the National Health System (1). Despite these efforts, the detection of sources of conflict and the design of programs adapted to the context where they are going to be applied remains a necessity to be met. To this effect, our hypothesis is that, through this qualitative study, we will be able to explore with a sufficient level of specificity which are the sources of conflict perceived by the professionals and how they consider these can be solved through specific measures for the improvement of the work environment. For this reason, the main objective of this study is to conduct focus groups with Primary Care professionals. Specifically, on the one hand, the aim is to explore the sources of conflict perceived by these professionals, emphasizing those that depend on their own work performance. On the other hand, we will delve into specific measures for these conflicts that could reduce PC conflict.

## Methods

### Setting

This study was approved by the ethics committee of the University of Murcia (ID: 3555/2021). For its development, qualitative methodology based on focus groups was applied. Specifically, a qualitative research design was proposed from the perspective of grounded theory with a constructivist approach (31). The research team that developed this study has extensive experience in the healthcare field and in research and in the publication of scientific articles on user violence toward health care personnel, both qualitative and quantitative. For the writing, the recommendations established for qualitative research in the COREQ guide (32) were followed and checked.

### Participants

The sample consisted of 44 workers from the Primary Care centers and management (68.2% were women). The mean age of the participants was 50.3 (SD = 7.95) with an age range between 38 and 64 years. All participants were of Spanish nationality, 27.27% of them were medical staff, 47.73% were nurses and 25% were administration or support staff. Those selected, at the time of the interviews, were in their current job for an average of 7.07 years (SD = 6.16) and their experience in the profession was 21.66 years on average (SD = 8.17) (Table 1).

**Table 1:** Summary of qualitative, sociodemographic and labor variables.

	***N***	**%**
**Sex**		
Male	14	31.8
Female	30	68.2
**Nationality**		
Spanish	44	100
Profession	12	27.3
Medicine	21	47.7
**Nursing**		
Administration and other	8	25
**Type of contract**		
Permanent	39	88.6
Fixed-term employment contract	5	11.4
**Performing other occupational activity related to the profession**		
Yes	6	13.6
No	38	86.4
**Conducting continuing education activities**		
Yes	21	47.7
No	23	52.3
**Work leave in the last 12 months**		
Yes	10	22.7
No	34	77.3

### Instruments Used

In order to explore the sociodemographic and occupational variables of our sample, an ad hoc questionnaire was administered. Specifically, the variables evaluated were: year of birth, sex, nationality, seniority in the current job, seniority in the profession, professional group, type of contract, performance of other activity related to the profession, continuing education activities, and sick leave in the last 12 months.

### Data Collection

Data were collected between December 2019 and March 2020 in collaboration with the Health Service of Murcia (SMS), located in southeastern Spain. Prior to contacting potential study participants, a collaboration agreement was made with the SMS and a script was prepared for the focus groups. This script was prepared by generating statements related to the theoretical dimensions extracted from the literature review and was complemented by *brainstorming* and concept mapping techniques in a group of experts (Table 2). Finally, it was tested in a pilot group that was not included in the results of this study (33, 34).

**Table 2:** Focus groups script.

**Issue**	**Example**
Problems derived from the organization	What can be the reasons, from the point of view of organization, coordination, characteristics of the system itself, climate… that cause conflict between the user and the health personnel within the health system. Please remember that we are also interested in how you would solve these problems.
Problems derived from the professional himself	Behaviors and attitudes that you think the health personnel may have or have had and that have generated conflict. Please remember that we are also interested in how you would solve these problems.
Problems derived from the user	Behaviors, attitudes or any action that you have seen in the users that cause conflict between the user and the health personnel within the health system. Please remember that we are also interested in how you would solve these problems.

A total of 8 focus groups of between 6 and 12 professionals were conducted. The groups were organized as follows: group (1) formed by management and occupational risk representatives; group (2) formed by representatives of labor unions and other representative bodies; group (3) formed by the area coordinators of the collaborating health centers; group (4) formed by center coordinators of medicine, nursing and administration; and, finally, groups (5–8) formed by workers in PC centers where there were medical, nursing and administrative professionals. Participants in groups 1–4 were interviewed at the central offices of the SMS. Participants in groups 5–8 belonged to 5 PC centers belonging to the SMS.

For recruitment, professionals were invited by email or telephone, giving them a brief description of the objectives of the study. Subsequently, they were sent an email with detailed information and informed consent. Prior to the interviews, they were reminded of the confidential nature of the interviews, the objectives of the study and informed consent was again requested, this time verbally. All interviews were audio-recorded and subsequently transcribed. For the anonymization of the data, codes assigned to each person were used to ensure confidentiality. At the end of the study, a detailed report was made available to the participants and feedback on the results was requested.

### Data Analysis

An inductive and constructivist approach was used for data analysis following Braun and Clarke's thematic analysis proposal. This method seeks to find and discover patterns within the information collected through the focus groups (35).

Following this method, the analysis of the information was conducted in six phases. In the first phase, the recordings of the focus groups were transcribed, thus forming the basis of the analyses, so that the authors could become familiar with the data collected. Each of the transcriptions was conducted and supervised by at least two of the authors of this study. In this phase, the researchers took notes on those ideas that could be useful in later phases. In the second phase, initial codes were generated to identify extracts from the data using an inductive or bottom-up method. This method starts from the raw data without attempting to fit them to a pre-existing theoretical framework or preconceived ideas of the researchers. The codes were discussed in pairs by all of the authors, proposing multiple coding if there was no consensus.

In the third phase, topics and subtopics were developed by grouping these codes generated in the previous phase. In addition, maps and tables were developed to facilitate thematic organization and the rejection of non-relevant codes. To generate the topics, the researchers opted for a constructivist perspective, exploring the topics that are latent in the information. From this perspective, broader assumptions, structures and/or meanings are accepted to support whatever is expressed in the data.

Once the topics and subtopics were obtained, in the fourth phase, a step backward was made in the process, reviewing the codes or even the extracts of information in order to check if they were consistent with the data obtained in order to make any necessary adjustments. A topic was considered relevant if it was present in at least four of the eight focus groups. As an exception, topics that were present in fewer groups could be raised if there was consensus among the researchers.

Finally, in the last two phases, the final topics were defined and named and a report was produced. At the conclusion of these phases, a clear and concise idea was obtained of what the main topics and their respective subtopics were, and what role each one played, generating a document with literal examples for the description and understanding of the results.

## Results

**Figure 1:**
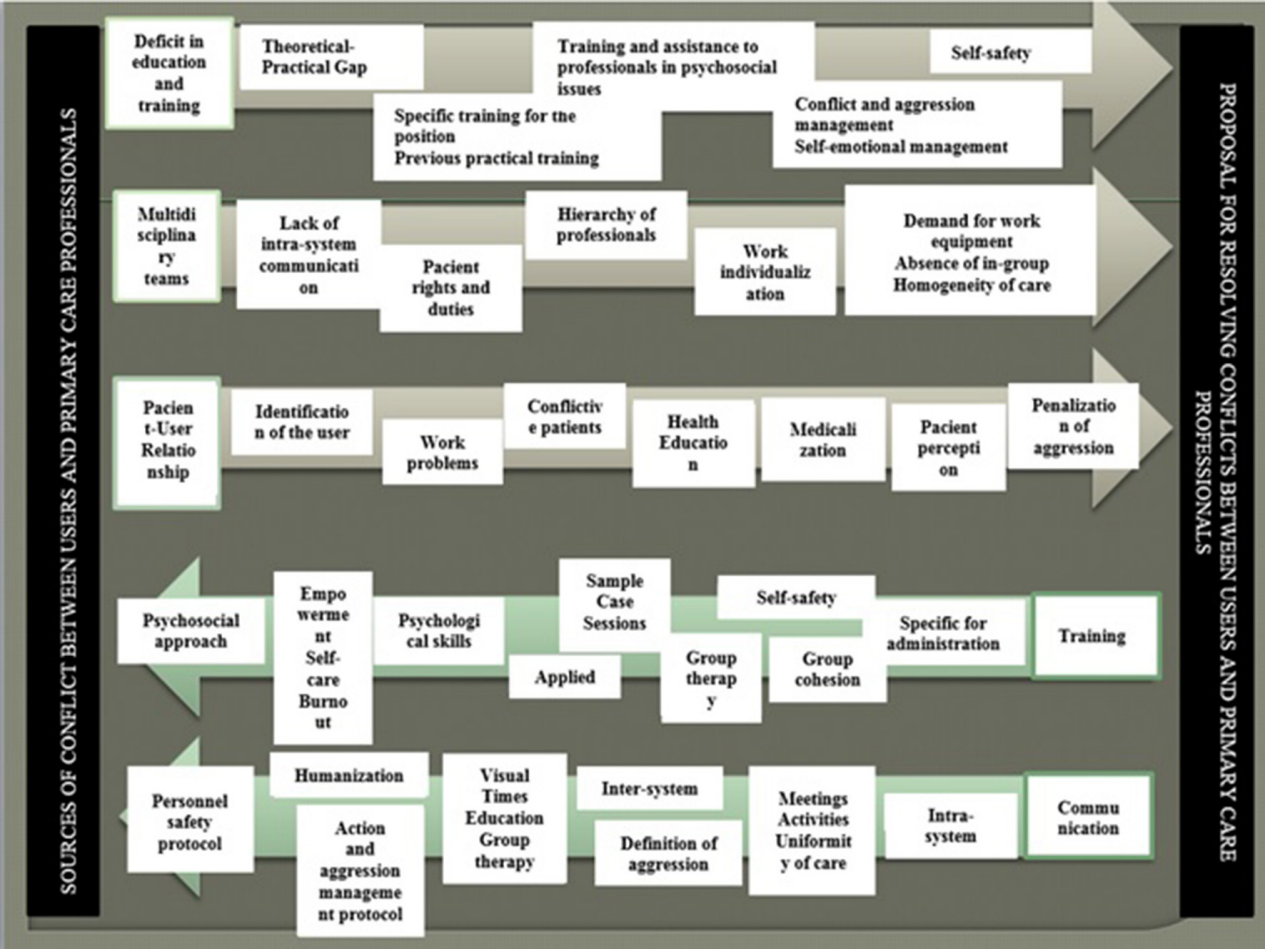
Sources of conflict and possible solutions in primary care centers from the professional's perspective: topics and subtopics.

The thematic analysis applied to the different focus groups identified a total of 3 thematic blocks related to focal points of conflict between PC users and PC workers. These were: (a) deficits in training or education, (b) the need to strengthen multidisciplinary teams, and (c) the patient-professional relationship. In addition, two thematic blocks related to ideas and proposals for solutions to the sources of conflict were also identified. These were: (a) training and (b) working to increase the quantity and quality of communication. Each of these blocks is further divided into subtopics as detailed below (see Figure 1).

### Sources of Conflict

#### Topic 1: Deficits in Training or Education

In all the focus groups, reference was made to the need for training of PC service workers. Specifically, more practical training, more information on the psychosocial approach, conflict management and personal safety were requested. Although this type of training has sometimes been offered, the professionals consider that it does not seem to be accessible to all the professionals in PC centers or is not provided to those who, in their opinion, need it:

“*What happens is that many times the courses are voluntary and the people who need it the least sign up”*.

“*The truth is that we have courses on prevention of aggressions, and we have had to suspend courses on prevention of aggressions due to the lack of enrollees”*.

“*There is a widespread ignorance by the professional of the community's catalog of resources”*

As mentioned, there seems to be a perception of deficits in theoretical-practical training regarding conflict, workplace violence, and user care skills. Interviewees report a need for job-specific practice-based training (e.g., administration), and they have the feeling that there is a deficit in practical training for future healthcare professionals in the faculties.

“*I was wondering (…) if the university curricula match the reality that is needed from professionals”*.

“*but they are told about diseases, never about sick people, never about people”*.

In addition, in all the groups, the workers' concern about the lack of psychosocial and emotional resources that would be useful both for their own well-being and for their ability to relate effectively with users was evident.

“*You are burned out and attack faster and cause conflict”*

“*I went home feeling awful for all the things they told me, I saw that, there, I had no strength whatsoever”*

“*I don't particularly consider myself psychologically prepared, and still what I don't… I'm not prepared, what I do is, well, calm down and do what the user wants me to do”*.

Finally, in this block, the feeling of lack of protection or helplessness that professionals feel in *certain situations* of conflict with users was highlighted.

“*I see myself quite unprotected”*

“*They enter the consult and you are totally unprotected, if someone wants to slap you, they can do it just fine”*

#### Topic 2: Deficits in Multidisciplinary Teams

A large number of participants alluded to the need to create or strengthen multidisciplinary work teams in PC centers. To this end, greater or better coordination and communication, hierarchical equality between the different professionals and the avoidance of individualization of work are demanded.

Regarding the deficits in coordination and communication, the professionals consider that this can annoy and/or confuse users. That is, a user may receive different messages from different professionals, sometimes contradictory. This can frustrate both users and professionals, causing violent situations.

“*Many times, professionals get into conflict. We are not clear on the concept of global team functioning”*.

“*Sometimes it's referred from your consult, like okay, it's your turn. You get to your consult and tell them to go and get done whatever they need to get done, or to find their nurse for whatever reason, or to go to the administration for whatever reason; and this referral is not well done. Then, that annoys them so much that, when they have been around the circuit for four laps now, the first person they see gets gored… to take them away”*.

In addition, professionals feel that they are not respected by some users, and this relationship is influenced by their professional category.

“*Although there is not as much respect as before, there is a lot of respect for the doctor, a little less for the nurse, a little less for the auxiliary…”*.

“*They have lost a lot of respect, and in particular respect, for example, for the figure of the doctor, who was in a… who was super high. The doctor is not treated with respect, they are treated informally from the start”*.

Reference was also made to an excessive individualization of care. In this regard, the perception of the absence of in-group and care among the professionals themselves was highlighted.

“*I think we make the mistake of making plots to deal with individual issues as if they were collective and that makes them not have a sense of belonging to the group, it demotivates them and burns them”*.

“*We, healthcare professionals, are in the business of taking care of the population that comes for help, but we don't take very good care of each other among the team”*.

“*If we [nurses] get together and talk about vaccines and no one else goes, the doctors are not going to know about the update, they are not going to refer patients to us to vaccinate them, and the administrative is not going to redirect them* [the patients] *well”*.

#### Topic 3: Patient-Professional Relationship

The last block referring to the conflicts detected has to do with the patient-professional relationship. In this line, reference is made to the fact that all the professionals have experienced some violent situation, regardless of the worker's professional category. The personnel themselves recognize that, sometimes, the care provided could be of a higher quality, recognizing themselves as precipitants of some situations of conflict.

“*Sometimes we don't take care of them in the desirable way because we don't have enough time”*.

“*We have a number of users who are pissed off because of us, let's not forget that. Not personally, but the organization”*.

“*I understand that in the same way that there is a rude patient, there is a rude professional, in the same way that there is a patient who brings their problems and their stories and stuff, there is a professional who is also rude”*.

In the interviews, reference was made to the time allocated to each consultation as unrealistic, causing overload, poor attendance and delays in care, which ultimately ends up causing conflicts with users.

“*It's just that 5–6 minutes per patient is exhausting, that's impossible to meet”*.

This situation could be facilitating depersonalized or dehumanized treatment by some professionals, resulting in deficits in communication and/or empathy, infantilization of the patient, complacency on the part of the workers when faced with demanding patients, avoidance of conflict and responsibility on the part of the professionals, among others.

“*When you don't bring your affectivity into the communication process, things go wrong”*.

“*The doctor is obsessed with the test, or any healthcare professional is obsessed with the test, and you don't look at the patient's face”*.

The professionals also consider that, regarding users, there is “*a very high percentage of consultations due to lack of knowledge”*, which could lead to inappropriate use of PC. In addition, reference is made on many occasions to the fact that, despite the fact that the rights and duties of users are written and accessible, users seem to have a lack of information on this regard. This situation favors the focus on professional-user interaction in favor of rights and, in *certain situations*, obviates the user's obligations.

“*the duties are written and published, but they are not internalized”*.

“*as patients and in relation to the conflicts that usually occur, many times, they come from fact that the patient does not know well neither their rights nor their obligations, so, from there, sometimes conflicts arise between professionals and patients”*.

Within the inappropriate use of PC, the use of the emergency system by users is particularly noteworthy. The participants in the study affirm that some users come to the center claiming that their consultation is serious with the possible intention of avoiding making an appointment and waiting to be seen.

“*I think the biggest conflict that occurs in health care centers is that, it is the patient who comes to you from the emergency room and the other one tells you: I have an appointment; how are you entering [the consult] before me? And they end up confronting each other, they end up confronting the professional and everyone ends up angry”*.

“*They seek immediacy… there they want immediacy; I want it now.“*

Another idea that was very present in the focus groups was the need to provide care for psychosocial problems in PC. In other words, professionals claim that some users instrumentalize the use of the service to avoid facing an external conflict. They believe, therefore, that the professional's tools are limited in these situations, and that it is necessary to address the problem rather than, for example, to process a sick leave.

“*the relationship with the company or that your contract is running out, so you solve it by going to the health care space asking for a leave through malingering, is another important source of conflict with professionals and patients”*.

“*You go back to work and you end up like before, so you tell them: But if you are going back to work and you are going back to the same conditions, wouldn't it be better to improve your work situation and then you won't need the sick leave?”*

### Proposed Solution for Sources of Conflict

#### Topic 1: Training

When asked about how they would address the deficits mentioned above, the professionals considered that a training offer adequate to the needs was necessary, this being the most repeated idea in all the focus groups. On the one hand, they pointed out the need to improve assistance by proposing training in group therapy, psychosocial approach to cases, demedicalization or classes/meetings about cases. On the other hand, the need to improve personal competencies in terms of self-security, skills in psychological techniques, empowerment, self-care, consequences of violence or communication skills and training for administrative staff was raised. Finally, reference was made to conflict management training to facilitate specific solutions such as applied training and mediation. For the interviewees, this training is perceived as essential, regardless of the professional group. According to the focus groups, this training should take place in the workplace in order to be effective, during working hours and in a supervised manner. In addition, it should be planned periodically and, if possible, taking into account that it is necessary to repeat this training offer for future incorporations to the service.

“*On-site and supervised training, for example, a strategy has been developed that I have found formidable. Motivational interviewing training for all professionals, that's great! If we work with that every day… well, it could be considered that if one of the company's objectives is to avoid… they would have to, as a matter of routine, encourage the training of their professionals”*.

“*I think the solution is to learn to control conflicts and communication strategies that are fundamental”*.

Specifically, with regard to training in group therapy, the professionals consider that this, among other issues, would serve to reduce the delay in care, as well as to approach the user and empower them, which is why training in this type of therapy is considered to be essential.

“*These functionally patients, that is, those who feel sick, but are not sick, are at risk of becoming chronic and could be treated, instead of one at a time, in a group”*.

“*elderly people, a group of… who are elderly and maybe they come here every now and then because they are alone, because they are whatever… that is better, that would be great for them, but for elderly people, for example, there are no groups”*.

In general terms, they refer to practical or applied training that would allow the professional to acquire real competencies in conflict situations. This training should be offered from the base of the profession and supervised, where the professional has feedback on their performance. This training should also include the approach to psychosocial problems which, although the professionals recognize that this is a function of other professionals such as psychologists or social workers, they consider necessary given the scarce or non-existent presence of these specialists in PC. Furthermore, they claim that the earlier this training is in the professional career, the better results it could provide.

“*It can be taken from the beginning, from the school and it should start from the faculty with the professionals”*.

“*MIR [Resident Medical Intern], when the reception is done, the reception training, that prevention normally intervenes in the part of health emergencies and all that, it could also be…it could be included there”*.

“*there are patients who come to us because indeed they feel bad, that the reason is not in their body so to speak, but they don't know it, they don't do it with a bad intention, they really… there is a dissociation between what… and well, then yes, with more training on your part in psychosocial discomfort or psychosocial well-being from a more comprehensive perspective that that does correspond to us, to primary and therapy, and we are very deficient for what we would really need”*.

Special mention should be made of the sessions proposed for training in conflictive patient typologies and self-safety. The aim of this training is to generate competencies that prepare the professional for these situations. In addition, they are proposed to be recorded in order to receive feedback or supervision on the performance carried out.

“*Everyone working in joint sessions, not only of the team but also clinical cases and situations that we can deal with”*.

“*We were even recorded, they put us in extreme situations, they put us on phone calls all the time while you were attending the user, and then you could see yourself, how you were acting, and how you changed your way of gesticulating”*.

“*There is a lack of training in self-security for healthcare professionals… in the placement of the consultation rooms, they should study well how to place the table… I always make sure there are two doors…”*.

The non-healthcare professionals interviewed consider that it is necessary to increase their competencies in management and user care, as can be seen in the following example:

“*Administrative people who are going to be dealing with people who are customers would be perfectly trained and educated on how to properly care for a patient”*.

The professionals made multiple references to the need to develop skills such as assertiveness, empathy or frustration management, among others. These skills are considered as an added value for work performance, helping to manage or avoid conflict situations.

“*I think we always lack a little bit of empathy as well”*.

“*We see people with Burnout Syndrome, or who are very burned out from care”*.

In particular, among these skills, the need to increase the communication skills of professionals, such as adapting language to each user, was very often repeated. This training is perceived to be more relevant for nursing and medical professionals.

“*communication skills in Medical School, as a subject, or a part within Psychology”*.

“*to teach us some techniques to act and say” no “to patients,” no “but in a way that doesn't assault me”*.

“*to try to explain to them, in a way that they understand, the reality of the information”*.

Finally, to promote group cohesion, meetings are proposed in which the objectives revolve around the elimination of hierarchies, encouraging respect and equality between professional categories.

“*It is very important to have respect, respect among professionals, that there are no hierarchies”*.

“*the fact that it is a relationship that is not so hierarchical, but that everyone is able to be given a voice and the voice is respected because everyone contributes”*.

#### Topic 2: Communication

This block refers to strategies that should be followed by the professionals themselves with the aim of improving intra-system communication (within the center itself) and inter-system communication (between different services, for example, PC and hospitals). The need to create a specific protocol for cases of aggression is also proposed, ranging from prevention to possible penalization of the user for the aggression. Finally, it is proposed to promote the humanization of the interaction with the patient and the health teaching function in the community.

“*that teamwork also helps a lot when it comes to organizing the circuit and organizing the functioning of the internal and external team”*.

“*to all agree on having an attitude and to all agree with that and support each other”*.

“*to start a campaign to improve the image in society, of the professional, of everything a professional has to do, of how they work every day, and how they work hard for the rest of the people. So, they don't think when they enter the consultation… so they see what is on the other side of the table, and that they regain that trust”*.

Regarding intra-system communication, it has been proposed the creation of periodical meetings and the formation of work teams inviting the rest of the colleagues of the service.

“*regular meetings with another group of professionals, who may not even have to be from the same health center, in order to maintain a certain independence”*.

“*insist that the teams, even if it is a topic that has nothing to do with the orderly in your opinion, invite them”*.

It was considered essential for the professionals to carry out actions aimed at making the real making visible and publicizing the real functioning of the PC. In this regard, they consider that these actions should be accompanied by Public Health information, both at a general and specific level, on the use of emergencies, group care and emotional and psychosocial education in PC. The right communication of the user's rights and duties is also considered important.

“*To work with patients, not only health care but other types of care at the community level, so that they can learn more about our services, which are sometimes unknown”*.

“*Health education has to start from childhood, self-care has totally disappeared…”*.

“*(…) in health centers, when you see the poster of rights and duties. There are 26 rights and 7 duties, and the duties are written in tiny letters. We are failing in that”*.

The creation of a welcome plan for people who do not know how PC works has been considered relevant. This protocol would be aimed at both users and new professionals.

“*They have a welcome program where they show the whole center and teach how to make an appointment; that leads to avoid conflicts”*.

“*(…) just like the patient you have to give them [the poster] and say 'Look, you come to this center and these are your rights and these are your duties and stuff'. To the workers as well, to make the welcome pack and say 'Hey, you are going to start working with us, but you need to take to this communication course ”*.

On the other hand, the professionals interviewed consider that their functions should go beyond the PC center itself, referring to the importance of communicating with the community, strengthening ties with other services and generating work teams with representatives of all these groups.

Regarding the aforementioned protocol on prevention and intervention in the face of aggression, there seems to be some ignorance of how to act and what is really considered an aggression. This is why they demand the creation of an action guide.

“*When there is an attempt of aggression you don't have any mechanism and that should be protocolized”*.

“*Handling conflictive situations. In other words, how to act when an angry patient arrives and, from the start, disrespects you”*.

“*The indicators of aggression are mostly subjective… so there is too much subjectivity and then it is complicated to really monitor the problems”*.

The concept of humanization of care was very frequently mentioned in the interviews. In this sense, it seems that professionals refer to the elimination of physical barriers between professional and user. In addition, they propose promoting involvement with the user so that they feel cared for and looked after by the system. Along these lines, they suggest improving the treatment given in waiting rooms or interacting with users, among other measures.

“*A doctor-patient or healthcare professional-patient relationship that is more open; apart from being a professional relationship, it has to be a relationship of trust”*.

## Discussion

In response to the objectives of the study, three topics were identified related to conflict sources in PC and two topics related to proposals for resolving or reducing these conflicts. The professionals identify deficits in the training received, asking for it to be more practical, with a psychosocial approach, to generate competencies in conflict management and personal safety. There is also a need to create or strengthen multidisciplinary teams, with good coordination and communication and hierarchical equality between the different professional groups. Finally, there is evidence of problems in the patient-professional relationship, with references to the high frequency of violence toward professionals due to the burden of care, delays in care, dehumanized treatment or misuse of PC by users. Regarding the proposed solutions, the professionals consider it appropriate to promote training in group care for users, psychosocial approach to cases favoring de-medicalization, classes on cases, self-security or self-care, among other proposals. They consider it necessary to promote internal communication, both within the center itself and externally, with other centers and the community itself. In addition, it is proposed to generate an aggression management protocol to unify criteria.

Although not many previous studies evaluating conflicts in PC have been located, results equivalent to those presented here have been identified in other health services (8, 36–43). Among those studies that aim to determine the working environment in PC, a recent study stands out that applies a methodology similar to this study, with the difference that the sources of conflict and proposed solutions are evaluated in PC users. Both studies coincide in the inappropriate use of the system due to the user's lack of knowledge, deficit in consultation times that generates conflict, the professionals' lack of communication skills, the users' misuse of emergencies, depersonalized treatment and deficit in the management of psychosocial problems in PC. Likewise, both parties also seem to agree on some proposals identified in our study, such as the creation of support groups to decongest waiting lists, professional training and more information for the user (29). These studies show the need to create preventive interventions in PC centers focused on the sources of conflict and the proposals for improvement shared by both users and professionals.

Specifically, this study shows a demand for training in group therapy to facilitate evaluations and/or interventions, considering this to be a way to improve the humanization of care, reduce waiting times, delays in care and/or workload. Regarding the approach to psychosocial problems, although they recognize that it would be more appropriate for this to be carried out by specialized professionals, such as a psychologist, since this figure is not available in PC, specific training is considered necessary for these cases. Fletcher et al. (44) observed an improvement in the professional-user relationship after the implementation of Mutual Help Meetings where users offer and receive mutual help and support. In our opinion, this could also be a way to work on psychosocial or occupational problems, obtaining a decrease in the delay in assistance by making use of support groups with users who share similar situations or pathologies. In addition, such proposals could foster and maintain the sense of community that is characteristic of PC, through which both staff and users take responsibility for addressing together the concerns they experience.

Regarding specific training in improving the work environment, the creation of meetings aimed at managing interpersonal relationships is perceived as necessary, especially in the case of internal hierarchies, promoting respect and equality among professionals. This type of approach has proven useful in previous studies. This training should also be oriented to conflict situations with users (29, 44–47). In this case, it should include skills for the care of conflictive patients, self-confidence, assertiveness, communication skills, empathy or frustration management in the face of aggression. It has been observed that professionals who participate in this type of training show a greater number of coping strategies and a greater perception of competencies to deal with violence, perceiving themselves as with greater ability to carry out a biopsychosocial approach to cases (48, 49). This type of training could promote staff confidence and facilitate the management of conflicts experienced during their professional career. Along these lines, a 2015 review of 127 studies found that occupational health and safety training should be carried out in blended learning modules, taking advantage of the convenience of online formats and the benefits of face-to-face training (50). The benefits of training seem to be shared by a large part of the scientific community. In general, it is considered that training should be multidisciplinary, working on aspects such as occupational health, industrial hygiene, safety management or ergonomics at work (51). Although it may be included in some of these aspects, we believe that workplace violence, especially in healthcare professions, deserves special attention in these training plans.

Previous studies have indicated that training for health professionals is useful when it is implemented on a continuous, applied basis and with direct feedback to the professional on their performance (16, 25), in accordance with what was proposed by the professionals in our results. Therefore, we consider that this training should be implemented as soon as possible in the professional career (preferably in the faculties), also favoring the biopsychosocial approach and based on practical cases. In this sense, it is observed that prevention programs composed of different training units with videos or case studies are a potentially effective way to present information on workplace violence, especially when there is less experience (16). The study by Maagerø - Bangstad et al. in 2020 describes that competence development activities delivered through a course on continuing education in violence risk assessment and management proved to be empowering both individually and as a group. A reduction in threatening behaviors and an increase in preventive and conflict management strategies were observed (16). Professionals' self-safety was also studied by Gamme and Erikson in 2018 (51). These authors consider that building confidence, security, tools to adapt to users' stress levels, as well as tolerating and accepting rejection from them, can promote a sense of achievement and growth. To this end, they propose as necessary to engage in conversations with users about personal experiences and to use theoretical knowledge along with common sense. Finally, although much of this training should be common for PC staff, specific meetings should be considered for the different professional categories, especially for those who perform functions that are far from the requirements for access to the position, such as administrative staff (37, 48, 52).

In our results, there is evidence of deficits both in the internal communication within the health system itself and in the communication with the community or users. Within the center itself, regular meetings or leisure activities are proposed to facilitate mutual respect and collaboration among the workers themselves. The professionals request coordination, communication, hierarchical equality between professional groups, distribution of the center according to work teams, joint and homogeneous work and group cohesion. It is considered that the respect perceived by users and colleagues is different depending on the professional category to which one belongs. They detect an excess of individualization in the work and a deficit of cooperation with other areas of the system (hospital, specialized care, etc.,) leading to a lack of consensus among the different professionals in the approach to the same case. Peer support in daily work is described as a decisive factor in managing secure relationships with users and improving staff communication and cohesion. In this sense, the need has been pointed out to create open and confidential spaces where colleagues can be guided and advised, openly discuss and make common plans or hold safety meetings in the face of violent situations, as well as favoring the creation of work teams, which would lead to greater cohesion, support, affection and empathy among workers and homogeneity in user care (16, 18, 53).

As mentioned above, during COVID-19 there has been an increase in the number of situations of stress to which health professionals have been subjected, with potentially serious consequences for their health. Among the main causes of this increase, the lack of information and transparency, the overload of care or the lack of material and personnel resources have been mentioned (9–12). Along these lines, the measures proposed by the participants in our study could be useful for improving the work climate in Primary Care centers even in exceptional times such as the present.

With regard to users, there are many factors that affect the patient-professional relationship in PC centers, such as emergency management, user identification, consultation times, delays in care, conflictive or demanding patients, etc. (8, 38, 39). As a general proposal, professionals consider it essential to “educate” the user in these aspects, in accordance with what has been observed in the literature (37, 48). Previous studies have reported the usefulness of the creation of community representatives and support groups to foster the professional-patient relationship. The role of these professionals would be to encourage conflict resolution by helping to foster and maintain a sense of community whereby both staff and service users take responsibility for addressing violence together (18, 36, 40).

Furthermore, in line with what has been observed in other studies, it is proposed to create a protocol that addresses all relevant aspects of aggression, from prevention to its penalization, taking into account tools for the humanization of the interaction with the patient and the healthcare teaching function (16, 29). In relation to this, Maagerø - Bangstad and colleagues considered that the content of these protocols should be oriented to information on the existence of instruments, mechanisms and formal flows of notification of violence at work and communicate activities and support structures for professionals involved in episodes of violence. These authors state that when staff become familiar with the user's identifiable signs of aggression, they can better implement interventions and calm aggressive users (16).

Although user violence has been extensively studied, no previous studies have been found that propose a prevention plan and prove its efficacy in PC services. In our opinion, this study can serve as a basis for the creation of these programs. It is important to know what the main problems perceived by the professionals are, but also how they consider the best way to solve them. We cannot ignore the fact that they have direct experience with their job and their opinion is essential both to maximize effectiveness and to ensure that the proposed plans are accepted and can be adapted to the needs and work rhythm of these professionals. In our opinion, although the efficacy of these proposals or their generalization needs to be tested, we consider that they increase the evidence available for the approach of this type of programs that could be implemented in both national and international services.

This study should be interpreted with the limitations that are characteristics in qualitative studies. These results are limited to the description of the information reported, and no further inference can be made. Furthermore, the sample was collected in a specific region of Spain, and the results may be characteristic of this population, so it would be interesting if this study could be replicated in other locations. Although the sample was small (*N* = 44), the results showed adequate saturation levels for qualitative research, and were clearly associated with some of the areas that have been previously studied as foci of conflict in previous literature. Finally, it is important to continue conducting studies on these and other possible sources of conflict in PC, as well as to obtain a greater number and variety of proposed solutions, since the variables reflected in this study may be insufficient for a complete approach to violence. The authors propose three types of studies for the future. On the one hand, longitudinal studies of a quantitative nature that will allow us to know in depth the relationship of the variables studied here with the aim of proposing explanatory models. On the other hand, the design of an intervention program with the solutions proposed here, with a control group, so that the efficacy of these solutions can be quantified. At the same time, it would be advisable to again find out the perception of the professionals on how the emergence of SARS–CoV-2 has influenced what has been reported here and the reception of the proposals for improving the climate and management of violence for this specific situation.

## Data Availability Statement

The raw data supporting the conclusions of this article will be made available by the authors, without undue reservation.

## Ethics Statement

The studies involving human participants were reviewed and approved by 3555/2021. The patients/participants provided their written informed consent to participate in this study.

## Author Contributions

DP and JR-H: conceptualization. DP: methodology, formal analysis, and investigation. CP-M, JR-G, and JR-H: validation. DP, CP-M, JR-G, and PL-R: writing original draft preparation. JR-H and BM-J: writing review and editing. BM-J: funding acquisition. All authors have read and agreed to the published version of the manuscript.

## Conflict of Interest

The authors declare that the research was conducted in the absence of any commercial or financial relationships that could be construed as a potential conflict of interest. The reviewer EP declared past co-authorship/collaboration with the author DP to the handling editor.

## Publisher's Note

All claims expressed in this article are solely those of the authors and do not necessarily represent those of their affiliated organizations, or those of the publisher, the editors and the reviewers. Any product that may be evaluated in this article, or claim that may be made by its manufacturer, is not guaranteed or endorsed by the publisher.
